# Burkitt-type lymphoma in France among non-Hodgkin malignant lymphomas in Caucasian children.

**DOI:** 10.1038/bjc.1982.107

**Published:** 1982-05

**Authors:** T. Philip, G. M. Lenoir, P. A. Bryon, R. Gerard-Marchant, G. Souillet, N. Philippe, F. Freycon, M. Brunat-Mentigny

## Abstract

In a retrospective analysis of 87 cases of Caucasian childhood non-Hodgkin malignant lymphoma (NHML) from Lyon, France, all the case were diffuse lymphomas, but 47 were diagnosed as monomorphic small non-cleaved NHML, pathologically indistinguishable from Burkitt's lymphoma (BL). BL could then be the most frequent childhood lymphoma in France. This homogeneous series allows better definition of the characteristics of BL within NHML. Age distribution is similar to that of endemic BL, with a sex ratio of 3.7/1. Abdominal masses are initially present in 68% of the cases, whereas jaw is involved in only 4%. The disease is characterized by its overwhelming evolution in the absence of therapy. However, complete remission (CR) is usually obtained after the first chemtherapy regimen. Most relapses occur at 3-8 months. Death could be related to cerebrospinal fluid (CSF) involvement, local recurrence or secondary marrow involvement. Ninety per cent of the patients alive with no evidence of disease (NED) 8 months after CR can be considered as definitely cured. Our study on Caucasian children with NHML indicates that, from histological and clinical criteria, nearly half the cases are very similar to African BL. Even though EBV rarely associated with our cases, BL could be a worldwide lymphoma.


*Br. J. Cancer* (1982) **45**, 670

### BURKITT-TYPE LYMPHOMA IN FRANCE AMONG NON-HODGKIN MALIGNANT LYMPHOMAS IN CAUCASIAN CHILDREN

T. PHILIP*, G. M. LENOIR†, P. A. BRYON‡, R. GERARD-MARCHANT§, G. SOUILLET||, N. PHILIPPE‡, F. FREYCON¶ AND M. BRUNAT-MENTIGNY*

*From the *Centre Léon-Bérard, Paediatric Oncology Unit, 28, rue Laennec, 69273 Lyon cédex 2, France, †International Agency for Research on Cancer, Lyon, ‡Hôpital Edouard-Herriot, Lyon, §Institut Gustave-Roussy, Villejuif Paris, ||Hôpital Debrousse, Lyon, ¶Hôpital Bellevue, St Etienne, France*

Received 8 December 1981 Accepted 28 January 1982

**Summary.—In a retrospective analysis of 87 cases of Caucasian childhood non-Hodgkin malignant lymphoma (NHML) from Lyon, France, all the case were diffuse lymphomas, but 47 were diagnosed as monomorphic small non-cleaved NHML, pathologically indistinguishable from Burkitt's lymphoma (BL). BL could then be the most frequent childhood lymphoma in France.**

**This homogeneous series allows better definition of the characteristics of BL within NHML.**

**Age distribution is similar to that of endemic BL, with a sex ratio of 3·7/1.**

**Abdominal masses are initially present in 68% of the cases, whereas jaw is involved in only 4%.**

**The disease is characterized by its overwhelming evolution in the absence of therapy. However, complete remission (CR) is usually obtained after the first chemtherapy regimen. Most relapses occur at 3–8 months. Death could be related to cerebrospinal fluid (CSF) involvement, local recurrence or secondary marrow involvement. Ninety per cent of the patients alive with no evidence of disease (NED) 8 months after CR can be considered as definitely cured.**

**Our study on Caucasian children with NHML indicates that, from histological and clinical criteria, nearly half the cases are very similar to African BL. Even though EBV rarely associated with our cases, BL could be a worldwide lymphoma.**

CONFUSION AND CONTROVERSY persist with regard to the eponym "Burkitt's lymphoma" (BL) which was used initially to refer to a clinical and epidemiological entity in African children (Burkitt, 1958, 1962). Burkitt's initial report described a tumour "involving the jaw in African children" and "dependent on climatic factors" and suggested "the implication of an infectious agent in its aetiology" (Burkitt, 1958, 1962). In 1964, Epstein *et al.* first described, in a cultured Burkitt-cell line, the herpes-type virus now known as the Epstein–Barr virus (EBV). In tropical Africa and in New Guinea, 96–97% of cases are associated with EBV (see: Klein, 1975; IARC, 1981) and African endemic BL (EBL) is now recognized as a clear epidemiological entity.

The question whether this lymphoma is unique and restricted to endemic regions of the world, or whether it can also be found elsewhere, arose with 3 reports in 1965–1966 (Dorfman, 1965; O'Conor *et al.*, 1965; Wright, 1966). The answer should have been provided by the plethora of reports from many countries, published between 1966 and 1970 (see: Philip *et al.*, 1980*b*; Lenoir *et al.*, unpublished). However, there was a tendency to contrast EBL with BL arising outside Africa (non-endemic BL), the latter being sporadic, mainly abdominal in presentation, and usually not EBV-associated (Dorfman,

1965; Cohen *et al.*, 1969; Hirshaudt *et al.*, 1973; Ablashi *et al.*, 1974; Levine & Cho 1974; Banks *et al.*, 1975).

The confusion and controversy existed because most of the reports did not take into consideration the clear conclusions formalized in 1969 by a World Health Organization expert committee (Berard *et al.*, 1969). The committee carried out a detailed and objective study of cases gathered from Africa and other countries, and concluded that BL is a clear, pathological entity which is found all over the world and has features distinct from those of other undifferentiated lymphomas and leukaemias. It recommended that the tumour be referred to as a "malignant lymphoma undifferentiated Burkitt type".

In large series of childhood non-Hodgkin malignant lymphomas (NHML) outside the endemic region, BL was reported to account for from 0% (Wollner *et al.*, 1980) to 45·6% (Gerard-Marchant *et al.*, 1982).

The purpose of our study was to conduct a retrospective analysis of 87 cases of NHML in children. Two of us (RGM and PAB) were asked to identify cases to which the WHO definition of BL could be applied; and we report here 47 cases of BL found in Caucasian children from Lyon, France, between 1965 and 1979. This homogeneous series makes it possible to define epidemiological and clinical features of BL in France. We hope that these data will stimulate new pathological studies of previously reported series, since BL appears to be a clear clinico-pathological entity within childhood NHML.

### MATERIAL AND METHODS

Between 1965 and 1979, 146 patients under 16 years of age were treated for NHML at the Centre Léon Bérard, Lyon (75 patients) and at other chemotherapy units in Lyon (71 patients). Since 36 cases with incomplete clinical records were excluded, 110 were reviewed (Philip *et al.*, 1980*b*). Non-Caucasian cases and patients for whom initial sections were not available were also left out; thus our analysis was finally carried out on 87 Caucasian patients. Sections were reexamined for 76 patients (average of 3 stained sections from each patient) and new sections were available for 11 cases (at least 7 sections per patient). Sections routinely stained with haematoxylin and eosin and embedded in paraffin were thus available for all the cases. New sections were stained with haematoxylin and eosin, periodic-acid–Schiff, May–Grünwald–Giemsa, Wilder silver reticulin impregnation and Giemsa by the Lennert technique.

*Histological data.*—Histological material from all 87 cases before treatment was reviewed independently by 2 of us, without knowledge of the clinical data.

R.G.M. reviewed the cases using the Kiel classification (Gerard-Marchant *et al.*, 1974). Details of this classification in childhood NHML are given in Table I. P.A.B. reviewed the cases using a modification of the Rappaport classification (Bryon *et al.*, 1981).

TABLE I.—*Classification of non-Hodgkin's malignant lymphoma in children according to the Kiel classification and the personal experience of R.G.M.*

- Lymphoblastic
  - Burkitt-type
  - With convoluted nuclei
  - Others
- Immunoblastic
  - Pure
  - Immunoplasmoblastic
- Other
  - Epithelioid
  - Lymphoplasmocytoid
  - Reticulosarcoma
- Unclassified

TABLE II.—*Classification of non-Hodgkin's malignant lymphoma in children according to the personal classification of P.A.B. (a modification of Rappaport's classification).*

- Lymphoblastic
  - With convoluted nuclei
  - Without convoluted nuclei
- Burkitt-type
  - Typical monomorphic, small non-cleaved
  - Monomorphic, small non-cleaved with larger cells
- Histiocytic (diffuse, large cells)
  - Non-cleaved (immunoblastic)
  - Cleaved
  - Mixed
  - Blastic
- Unclassified

TABLE III.—*Clinical staging systems used in the present study*

| | Stage I | II | III | IV |
|---|---|---|---|---|
| Murphy | Single tumour (extra-nodal) or single anatomic area (nodal), with the exclusion of mediastinum or abdomen. | Single tumour (extra-nodal) with regional node involvement.<br>Two or more nodal areas on the same side of the diaphragm.<br>Two single (extranodal) tumours with or without regional node involvement on the same side of the diaphragm<br>A primary gastro-intestinal-tract tumour, usually ileocaecal, with or without involvement of associated mesenteric nodes only. | Two single tumours (extra-nodal) on opposite sides of the diaphragm.<br>Two or more nodal areas above and below the diaphragm.<br>All the primary intrathoracic tumours.<br>All extensive primary intra-abdominal disease.<br>All paraspinal or epidural tumours. | Any tumours already mentioned with initial CNS and/or marrow involvement. |

| | Stage A | AR | B | C | D |
|---|---|---|---|---|---|
| Ziegler | Single extra-abdominal site | Intra-abdominal tumour with > 90% surgically resected. | Multiple extra-abdominal sites | Intra-abdominal tumour | Intra-abdominal tumour with involvement of ⩾ 1 extra-abdominal site. |

Details of this classification for children are given in Table II.

Cases over which there was disagreement were reexamined, when complete clinical and follow-up information was made available to the pathologists. When both reviewers agreed on the diagnosis of BL, the case was included in the final series.

*Clinical data.*—The cases considered to be BL were first studied on the basis of a complete pretreatment evaluation, *i.e.*, complete blood count, measurements of serum urea, uric acid, electrolytes, serum lactic dehydrogenase, bilirubin, alkaline phosphatase and transaminase, marrow aspirate (1–4 per patient. average 2), urine analysis, i.v. pyelogram, chest roentgenograms, examination of cerebrospinal fluid for malignant cells and protein, and careful clinical examination.

Staging was done using Murphy (1977) and Ziegler (1977) classifications (Table III). In cases of localized BL (Murphy, Stages I and II; Ziegler, Stages A and AR) lymphography has been performed since 1975 to avoid errors of staging.

Before 1973, patients were treated with radiotherapy at the initial site and with a mono- or bi-chemotherapeutic regimen (cyclophosphamide and vincristine) for 2 years (33 patients). After 1973, patients were treated with polychemotherapy (Gout-Lemerle *et al.*, 1976), with a combination of cyclophosphamide, vincristine, prednisone and adriamycin (COPAD) (14 patients). Patients were judged to be in complete remission (CR) if there was no clinically palpable tumour and if all previously abnormal parameters had returned to normal. Survival curves, using the 2 staging classifications, were calculated by the method of Kaplan and Meier (1958).

### RESULTS

### *Histopathology*

The 2 pathologists examining histological material without knowledge of the clinical data agreed over 71/87 cases (81·6%); they agreed initially that 43 of these 71 were BL.

Results of the second review (in the light of clinical data and of the other reviewer's opinion) are summarized in Table IV. Agreement was reached to discard 4 cases that were not considered

TABLE IV.—*Study of 87 non-Hodgkin malignant lymphomas: distribution of histopathological types according to 2 reviewers (final opinion)*

| Kiel classification (R.G.M.) | | Total | % (of total of 83) | Modification of Rappaport's classification (P.A.B.) | | Total | % (of total of 83) |
|---|---|---|---|---|---|---|---|
| Lymphoblastic Burkitt-type | | 51 | 61·4 | Burkitt-type | | 47 | 56·6 |
| Lymphoblastic | | | | Lymphoblastic | | | |
| with convoluted nuclei | 9 | 14 | 16·9 | with convoluted nuclei | 9 | 15 | 18·1 |
| without convoluted nuclei | 5 | | | without convoluted nuclei | 6 | | |
| Immunoblastic | | 4 | 4·8 | Histiocytic | | | |
| | | | | non-cleaved | 5 | 6 | 7·2 |
| | | | | mixed | 1 | | |
| Unclassified | | 14 | 16·9 | Unclassified | | 15 | 18·1 |
| Not NHML | | 4 | | Not NHML | | 4 | |

Total of 87: Final agreement 83/87 95·4%.

to be NHML (the percentages are thus calculated on the basis of the 83 cases of confirmed NHML) and to consider the 83 remaining cases as diffuse lymphomas. With regard to the 4 cases on which there was final disagreement, they were considered as BL by R.G.M., and as lymphoblastic non-convoluted, histiocytic non-cleaved, mixed histiocytic, and unclassified by P.A.B.

The data can be summarized as follows:

(1) After discussion, 47 cases were diagnosed as BL. The 2 pathologists agreed that, in Giemsa-stained sections, BL is a diffuse lymphoma composed of monomorphic lymphoid cells of uniform maturity with a round nucleus and 3–4 nucleoli; they have a moderate amount of cytoplasm, which is well defined, deeply basophilic and usually contains clear vacuoles.

(2) One of us (P.A.B.), using a histomorphometric technique (Bryon *et al.*, 1981), identified 2 types of cell in the BL group: 30 cases with a typical, monomorphic, small non-cleaved cell, and 17 cases with a slightly larger cell. No differences were found between the 2 groups with regard to age, staging, initial presentation or survival (Philip *et al.*, 1980*b*).

*Clinical data*

The results obtained from the 47 cases are summarized in Figs. 1–4, and Tables V and VI.

*Sex and age distribution*

There were 37 males and 10 females among the patients (ratio 3·7:1).

The age distribution of the patients is given in Fig. 1, indicating a peak incidence between the ages of 6 and 9 (18/47 patients). The distribution between 2½ and 16 years shows an average for the whole group of 8·5 years.

*Initial presentation*

As shown in Table V, by far the most frequent site at presentation (in 68% of cases, 32 patients) was the abdomen. Lymph-node involvement was seen initially in 9 patients (19·1%) (7 with head

TABLE V.—*Site of initial presentation and stage (Murphy classification) in 47 cases of Burkitt's lymphoma in Caucasian patients*

| | Site | | | | | Stage | | | |
|---|---|---|---|---|---|---|---|---|---|
| | Abdomen | Lymph nodes | Subcutaneous | Intracranial | Jaw | I | II | III | IV |
| No. | 32 | 9 | 2 | 2 | 2 | 7 | 7 | 26 | 7 |
| % | 68 | 19·1 | 4·3 | 4·3 | 4·3 | 14·9 | 14·9 | 55·3 | 14·9 |

TABLE VI.—*Sites of relapse finally responsible for death in 28 cases (19 are alive NED)*

| First site of relapse or progression | Follow-up in months: 0 | 1 | 2 | 3 | 4 | 5 | 6 | 7 | 8 | 9 | 10 | 11 | 12 | 16 |
|---|---|---|---|---|---|---|---|---|---|---|---|---|---|---|
| Cerebrospinal fluid | | ●● | ●● | ● | ● | | | | | | | | | |
| Local recurrence | | | ● | ●● | ● | | ●● | ● | ●● | | | | | |
| Marrow involvement | | | ● | ● | ●● | ● | ● | | | | | | | ● (16 months) |
| Other sites | | | | | ● lymph node | | | ● chest | | | | | | |
| Other causes of death (at induction of therapy) | ●●●● | | | | | | | | | | | | | |

FIG. 1.—Age distribution of the 47 cases of Burkitt's lymphoma.

and neck localization) and other sites in 6 patients; *viz.* s.c. (2), intracranial (2), and jaw (2). Jaw localization thus accounted for only 4·3% of the cases in 2 boys (7½ and 8 years old).

### *Staging*

Two major classifications made it possible to distinguish 2 groups of patients. *Localized disease* (Murphy Stages I and II, Ziegler Stages A and AR) was seen initially in 14 patients (29·8%). This group of patients clearly has a good prognosis in both staging systems (*c.f.* Figs. 2 & 3). *Extensive disease* (Murphy Stages III and IV; Ziegler Stages C and D) was seen initially in 33 patients (70·2%); 7 of these (14·8% of 47 patients) had presented initially with marrow involvement (5–25% of Burkitt cells in marrow smears). This group clearly had a bad prognosis with both staging systems (*cf.* Figs. 2 & 3).

FIG. 2.—Percentage survivors typed by the Murphy classification * under treatment Non Evidence of Disease (NED); ○, out of treatment NED; ▲, alive under treatment in 2nd CR NED.

FIG. 3.—Percentages of survivors typed by the Ziegler classification (Stage B, with only 2 patients, has been omitted). Symbols as in Fig. 2.

FIG. 4.—Survival curve of all 47 cases of BL Symbols as in Fig. 2.

*Evolution*

Several points should be noted. Complete remission (CR) was not obtained for 13 patients; 2 died immediately after surgery for biopsy; 2 died during the first week due to metabolic disturbances; and 9 did not respond to therapy. Of the whole group, 40·4% of the patients are still alive (Fig. 4). When the 13 patients who did not go into remission are excluded, the survival of the remainder reaches 52·9%. Achievement of CR is thus a major factor for a good prognosis.

Two kinds of relapse can occur during treatment (Table VI). CSF involvement is the early complication, especially in patients with extensive disease, and occurred in 6/29 cases at 1–4 months of evolution. Later, there may be local recurrence (9/29) and secondary marrow involvement (8/29). Other sites of relapse were rare (1 in lymph nodes and 1 in the chest).

The survival curve was very different from that of other NHML in children (Fig. 4). Most fatal relapses occurred before 8 months of evolution even though some of the patients who relapsed before 8 months died later (1 after 20 months, 1 after 4 years). Twenty patients were alive with no evidence of disease (NED) 8 months after achieving CR, and 18 are still alive and can be considered cured. Two relapsed with marrow involvement 16 and 32 months after diagnosis (Table VI): 1 is dead, and 1 is still alive NED 24 months after relapse. Patients alive NED 8 months after CR, have a 90% chance of complete cure.

### DISCUSSION

Our data show that Burkitt-type lymphoma (BL) is a clear pathological entity within NHML in Caucasian children. Two of us, using different major classifications, agreed immediately on a diagnosis of BL for 43/47 patients (>90% of the final series). BL accounted for 56·6% of our 83 cases. The fact that this value is higher than in other series reported in the literature (Garwicz *et al.*, 1974: 19%—Sullivan, 1978: 25%—Cossman & Berard, 1980: 33%—Meadows *et al.*, 1980: 25%—Wollner *et al.*, 1980: 0%—Gerard-Marchant, 1981: 45·6%) may result principally from the recruitment of our patients; *i.e.* from a regional cancer institute where only solid tumours (never leukaemias) are treated. Lymphoblastic or non-abdominal lymphomas in children frequently present as leukaemias and are usually received by other hospitals in the area, whereas cases of BL, which is most frequently a solid abdominal tumour, are usually sent to the Centre Léon Bérard.

It is quite surprising to read the recent report by Wollner *et al.* (1980) of 35 abdominal lymphomas in children, indicating that none of them was a BL. It is our opinion that Berard's report that BL accounts for one third of childhood NHML outside Africa (Cossmann & Berard, 1980) is more likely to be epidemiologically correct than are our data. We (Lenoir *et al.*, unpublished) have found 635 cases of BL reported in the literature that should be considered to be non-endemic BL. The present report represents 7% of the cases published in the literature. It is our opinion that most of the abdominal lymphomas reported as "undifferentiated lymphomas" by the classification of Rappaport, could be in fact true BL; our data are not exceptional, and BL must be distinguished pathologically from other abdominal NHML in children.

These data also show that Burkitt-type lymphoma is a clear clinical entity. The age–incidence curve, which indicates a peak before the age of 10 years, is similar to that of the recent Olweny report from Uganda (Olweny *et al.*, 1980) and to other reports from different parts of the world (Lenoir *et al.*, unpub.). The finding in the present series that BL is mainly (68%) an abdominal lymphoma confirms previous reports from outside Africa (Dorfman, 1965; Hoogstraten, 1967; Arseneau *et al.*, 1975; Levine *et al.*, 1982). Other initial sites of presentation were also found, especially lymph-node involvement, but jaw tumours were found in only 2 cases, whereas 72% of patients described in Uganda showed a jaw swelling (Olweny *et al.*, 1980). There is thus a clear clinical difference between African and non-African BL. Nevertheless, BL should not be a synonym for "jaw tumour", as numerous authors have assumed, who identified BL only when a jaw tumour was present (Willemin *et al.*, 1966; Cuevas *et al.*, 1976; Joncas & Rioux, 1977). This assumption represented a major bias in the diagnoses of the 625 cases cited above (Lenoir *et al.*, unpub).

From our data, the characteristics of BL outside Africa are summarized in Table VII. We have found that patients alive NED 8 months after CR can be considered to have a 90% chance of complete cure. This short evolution suggests that non-endemic BL could be used as a model for therapeutic research. In our opinion, such research should be carried out primarily on the 3 principal sites of complication (CSF, local recurrence and marrow). One of the most interesting avenues of research in BL is the use of intensive chemotherapy followed by marrow autografts (Ziegler, 1977; Appelbaum *et al.*, 1978). Recent observations by our group (unpublished) confirm the high efficiency of this regimen even in patients in a second CR.

BL is thus a clinico–pathological entity in Caucasian children. It is also an immunological entity; when 10 of our 47 cases were studied by immunological methods, all tumour cells were found to be B cells with surface immunoglobulins. This finding is confirmed by all the data in the literature (Mann *et al.*, 1976). BL is also a cytogenetic entity: in 4 of our cases, the tumour cells were studied by banding techniques, and 3 translocations of (8;14) and one variant translocation, t (8;22), were found (Bertrand *et al.*, 1981). The 8;14 translocation, initially reported in African cases as a 14q+ (Manolov & Manolova, 1972), has been found in most non-endemic BLs (Zech *et al.*, 1976). Two variant translocations, t (2;8) and t (8;22), were then described in both endemic and non-endemic BL (Bernheim *et al.*, 1981). These translocations are now considered to be a characteristic feature of BL in all parts of the world. Finally, whereas 96% of endemic cases are EBV-associated, an association rate of only 10–20% was found in this series (Philip *et al.*, 1980*a*; Lenoir, unpub.).

It must be emphasized that the present study is strictly pediatric. Levine's studies in the US (Levine *et al.*, 1982) clearly show that BL is not only a pediatric lymphoma, and unpublished data by members of our group confirm this conclusion.

The only major differences apparent between non-endemic and endemic BL are the high incidence, the high rate of EBV association and the high jaw-tumour

TABLE VII.—*Summary of characteristics of non-endemic Burkitt-type lymphoma*

1. In Western countries, BL represents at least one third of childhood NHML.
2. The primary site of the tumour is usually abdominal or pelvic.
3. The disease is characterized by acute evolution in the absence of therapy.
4. The immediate risk, especially for extensive disease, is CFS involvement.
5. The second risk is local recurrence or secondary marrow involvement.
6. The evolution of the disease is short, 90% of relapses being $<8$ months from diagnosis.
7. Children alive NED after 12 months are probably cured.

frequency of the latter. The relationship between incidence and EBV association should be considered to favour the role of EBV in the pathogenesis of African BL. Our report of 100% EBV association in Arab Algerian children from a non-endemic area may indicate, however, that socio-economic level is more important than incidence or geography as an explanation of the EBV association (Philip *et al.*, 1980*a*).

BL is first and foremost a histopathologically distinct lymphoma. A retrospective analysis should now be made of large series of pathological specimens in order to establish the actual incidence of BL among childhood NHML. The term "Burkitt-type lymphoma" should be used in all classifications.

### REFERENCES

ABLASHI, D. V., DE-THE, G., EASTON, J. M., LIABEUF, A., LEVINE, P. H. & ARMSTRONG, G. R. (1974) Antibodies to Epstein–Barr virus (EBV) antigens in sera of American Burkitt's lymphoma patients. *Biomedicine*, **20**, 288.

APPELBAUM, F., HERZIG, G., ZIEGLER, J., GRAW, R., LEVINE, A. & DEISSEROTH, A. (1978) Successful engraftment of cryopreserved autologous bone marrow in patients with malignant lymphoma. *Blood*, **52**, 85.

ARSENEAU, J. L., CANELLOS, G. P., BANKS, P. M., BERARD, C. W., GRALNICK, H. R. & DE VITA, V. T. (1975) American Burkitt's lymphoma: A clinico–pathological study of 30 cases. I. Clinical factors relating to prolonged survival. *Am. J. Med.*, **58**, 314.

BANKS, P. M., ARSENEAU, J. C., GRALNICK, H. R., CANELLOS, G. O., DE VITA, D. T. & BERARD, C. W. (1975) American Burkitt's lymphoma: A clinico-pathological study of 30 cases. II. Pathologic correlation. *Am. J. Med.*, **58**, 322.

BERARD, C. W., O'CONOR, G. T., THOMAS, L. B. & TORLONI, H. (1969) WHO memoranda: Histopathological definition of Burkitt's tumour. *Bull. WHO*, **40**, 601.

BERNHEIM, A., BERGER, R. & LENOIR, G. (1981) Cytogenetic studies on African Burkitt's lymphoma cell lines: t(8;14), t(2;8) and t(8;22) translocations. *Cancer Genet. Cytogenet.*, **3**, 307.

BERTRAND, S., BERGER, R., PHILIP, T. & 6 others (1981) Variant translocation in a non-endemic case of Burkitt's lymphoma: t(8;22) in an Epstein–Barr virus negative tumour and in a derived cell line. *Eur. J. Cancer*, **17**, 577.

BRYON, P. A., FELDMAN, P., BERGER, F., GENTILHOMME, O., PHILIP, T. & FIERE, D. (1981) Lymphomes malins non-Hodgkiniens. Reconnaissance et classification morphologiques. In *Syndromes Lymphoproliferatifs* (Ed. Boiron) Paris: Masson. p. 13.

BURKITT, D. (1958) A sarcoma involving the jaw in African children. *Br. J. Surg.*, **46**, 218.

BURKITT, D. (1962) A children's cancer dependent on climatic factors. *Nature*, **194**, 232.

COHEN, M. H., BENNETT, J. M. & BERARD, C. W. (1969) Burkitt's tumour in the United States. *Cancer*, **23**, 1259.

COSSMAN, J. & BERARD, C. W. (1980) Histopathology of childhood non-Hodgkin's lymphomas. In *Non-Hodgkin's Lymphomas in Children*, (Ed. Grahampole), New York: Masson. p. 13.

CUEVAS, G., SILVAN, A., CANTON, G., DELGADO, J. & GUERRERO, D. (1976) Linfoma de Burkitt: Apportacion de un caso. *Ann. Esp. Pediat.*, **4**, 104.

DORFMAN, R. F. (1965) Childhood lymphosarcoma in St. Louis, Missouri, clinically and biologically resembling Burkitt's tumour. *Cancer*, **18**, 418.

EPSTEIN, M., ACHONG, B. & BARR, Y. (1964) Virus particles in cultured lymphoblasts from Burkitt's lymphoma. *Lancet*, i, 702.

GARWICZ, S., LANDBERG, T. & AKERMAN, M. (1974) Malignant lymphomas in children: A clinico–pathologic retrospective study. II. Non-Hodgkin's lymphomas. *Acta Paediat. Scand.*, **63**, 679.

GERARD-MARCHANT, R., JAMLIN, I., LENNERT, K., RILKE, F., STANFIELD, A. G. & VAN UNNIK, J. A. (1974) Classification of non-Hodgkin malignant lymphoma. *Lancet*, i, 406.

GERARD-MARCHANT, R., BAYLE, C. & CAILLOU, B. (1982) Histocytologie des lymphomes malins non-Hodgkiniens de l'enfant (Etude de 161 cas typés selon la classification de Kiel). *Arch. Anat. Pathol. Paris* (in press).

GOUT-LEMERLE, M., RODARY, C. H. & SARRAZIN, D. (1976) Le traitement des lymphomes malins non-Hodgkiniens de l'enfant. *Arch. Fr. Pediatr.*, **33**, 537.

HIRSHAUDT, Y., COHEN, M. H. & STEVENS, D. A. (1973) Epstein–Barr virus antibodies in American and African Burkitt's lymphoma. *Lancet*, ii, 114.

HOOGSTRATEN, J. (1967) Observations on Burkitt's tumour in central and northern Canada. *Int. J. Cancer*, **2**, 566.

INTERNATIONAL AGENCY FOR RESEARCH ON CANCER (1981) Burkitt's lymphoma programme, *I.A.R.C. Ann. Rep. 1981*, p. 85.

JONCAS, J. H. & RIOUX, E. (1977) Burkitt's lymphoma of the jaw in a Canadian child. *Can. Med. J.*, **117**, 367.

KAPLAN, E. L. & MEIER, P. (1958) Non parametric estimation from incomplete observations. *J. Am. Stat. Assoc.*, **53**, 456.

KLEIN, G. (1975) Studies on the Epstein–Barr virus genome and the EBV determined nuclear antigen in human malignant disease. *Cold Spring Harbor Symp. Quant. Biol.*, **39**, 783.

LEVINE, P. H. & CHO, B. R. (1974) Clinical features of North American cases. *Cancer Res.*, **34**, 1219.

LEVINE, P. H., KAMARAJU, L. S., CONNELLY, R. R. & 5 others (1982) The American Burkitt's lymphoma registry: Eight years' experience. *Cancer*, **49**, 1022.

MANN, R. B., JAFFE, E. S., BRAYLAN, R. C. & 4 others (1976) Non-endemic Burkitt's lymphoma: A B cell tumor related to germinal centers. *N. Engl. J. Med.*, **295**, 685.

MANOLOV, G., & MANOLOVA, Y. (1972) Marker band in one chromosome 14 from Burkitt's lymphoma. *Nature*, **237**. 33.

MEADOWS, A. T., JENKIN, R. D. T., ANDERSON, J. & 8 others (1980) A new therapy schedule for pediatric non-Hodgkin's lymphoma toxicity and preliminary results. *Med. Pediatr. Oncol.*, **8**, 15.

MURPHY, S. B. (1977) Prognostic features and obstacles to cure of childhood non Hodgkin's lymphoma. *Sem. Oncol.*, **4**, 265.

O'CONOR, G. T., RAPPAPORT, H. & SMITH, E. B. (1965) Childhood lymphoma resembling Burkitt's tumour in the United States. *Cancer*, **18**, 330.

OLWENY, C., KATUNGOLE-MBIDDE, E., OTIR, D., LWANGA, S., MAGRATH, I. T. & ZIEGLER, J. L. (1980) Long term experience with Burkitt lymphoma in Uganda. *Int. J. Cancer*, **26**, 261.

PHILIP, T., LENOIR, G. M., BRUNAT-MENTIGNY, M. & 4 others (1980*a*) Individualisation pathogénique du lymphome de Burkitt en France. Quel est le problème? Comment le résoudre? Résultats préliminaires. Rôle des anomalies cytogénétiques. *Pédiatrie*, **35**, 659.

PHILIP, T., BRYON, P. A., PHILIPPE, N. & 4 others (1980*b*) Individualisation anatomo–clinique du lymphome de Burkitt en France: A propos de 51 cas personnels. *Pédiatrie*, **35**, 677.

SULLIVAN, M. (1978) Therapeutic strategies for childhood lymphoma. In *Research Report of the University of Texas*. Houston: System Cancer Center. p. 425.

WILLEMIN, G., LAVAROC, M., MENUT, G. & 4 others (1966) Sarcome de type Burkitt chez un enfant européen ayant fait un séjour en Afrique. *Arch. Fr. Pediatr.*, **13**, 1183.

WOLLNER, N., WATCHEL, A., EXELBY, P. & CENTORE, D. (1980) Improved prognosis in children with intra-abdominal non-Hodgkin's lymphoma following $LSA_2L_2$ protocol chemotherapy. *Cancer*, **45**, 3034.

WRIGHT, D. (1966) Burkitt's tumour in England: A comparison with childhood lymphosarcoma. *Int. J. Cancer*, **1**, 503.

ZECH, L., HAGLUND, U., NILSSON, K. & KLEIN, G. (1976) Characteristic chromosomal abnormalities in biopsies and lymphoid cell lines from patients with Burkitt and non-Burkitt's lymphoma. *Int. J. Cancer*, **17**, 45.

ZIEGLER, J. L. (1977) Treatment results of 54 American patients with Burkitt's lymphoma are similar to the African experience. *N. Engl. J. Med.*, **297**, 75.


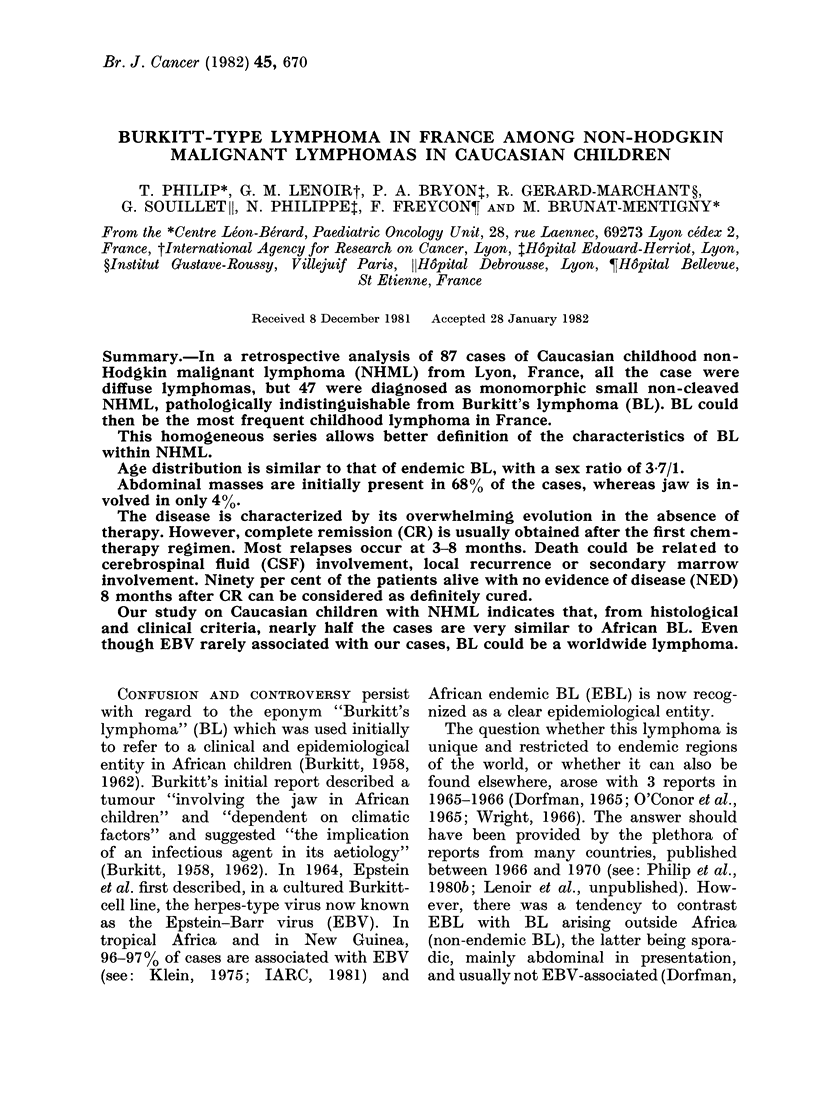

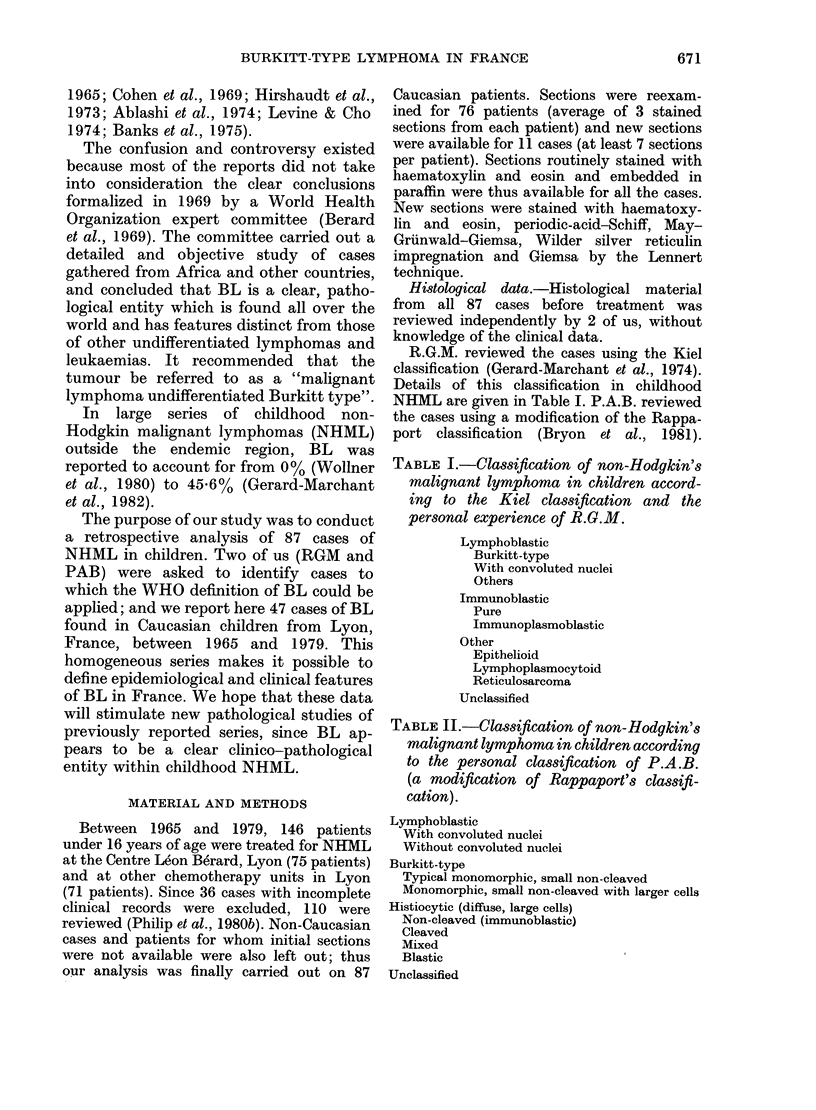

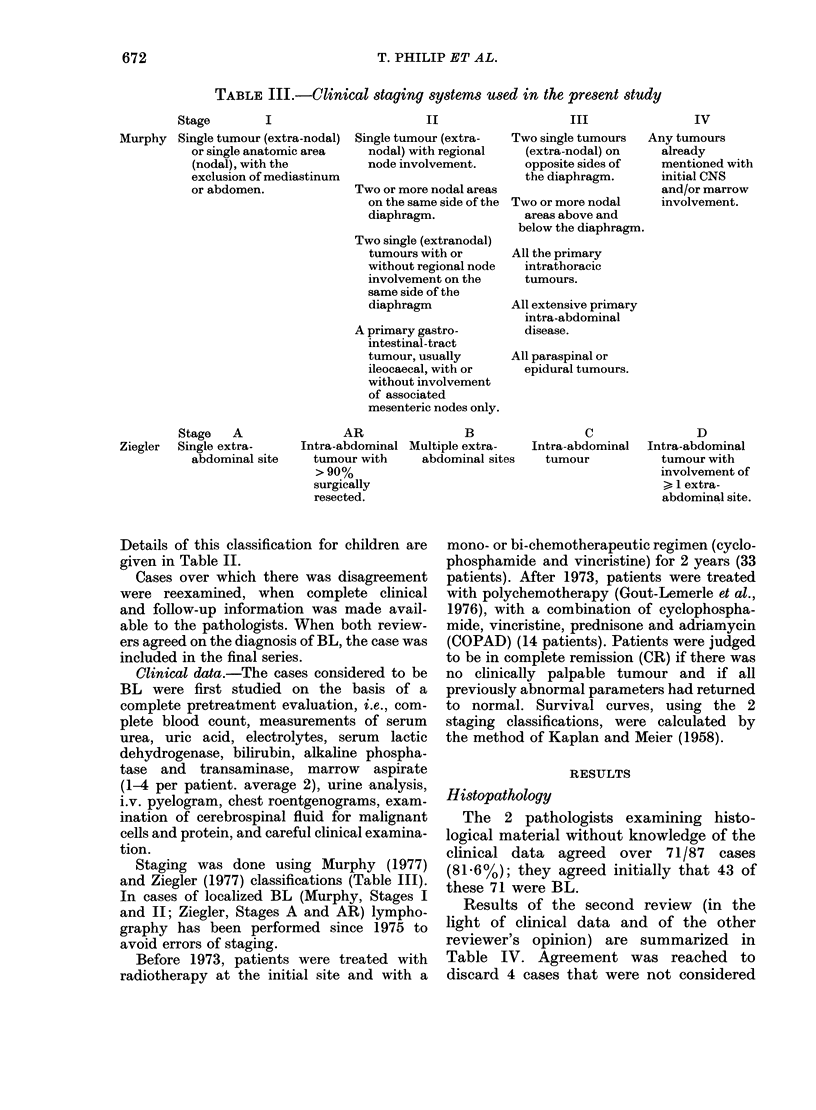

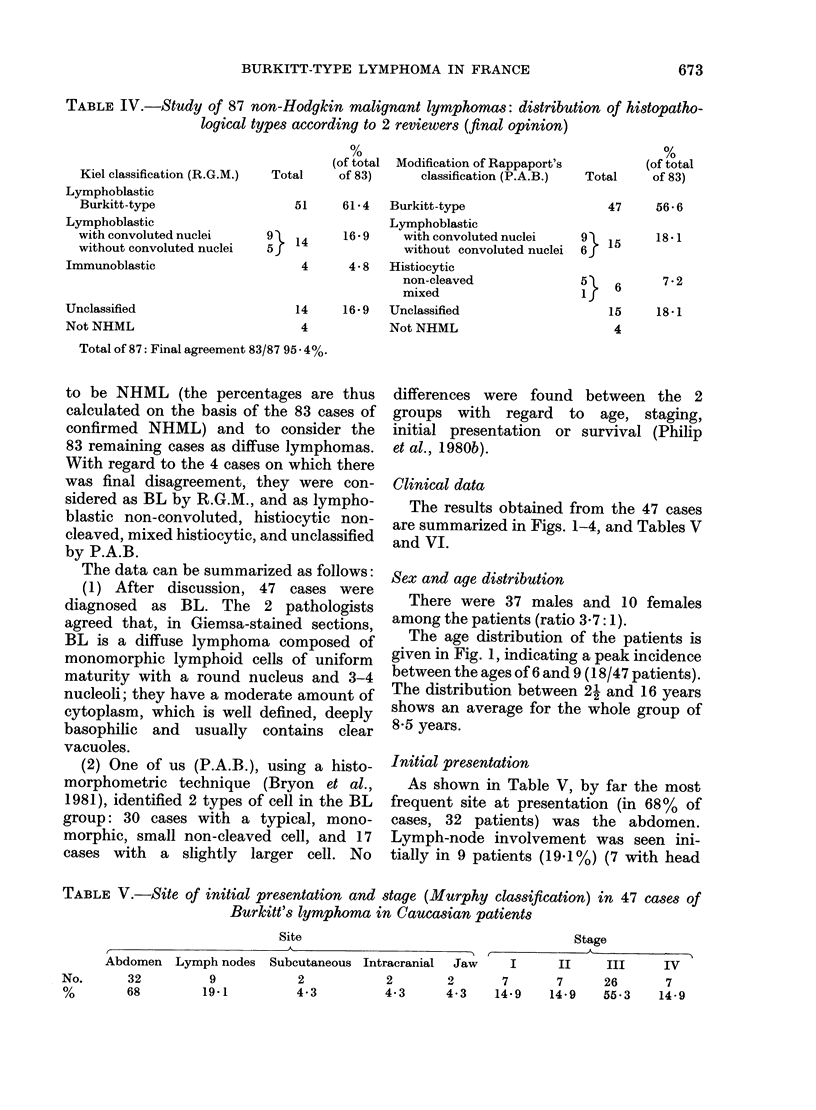

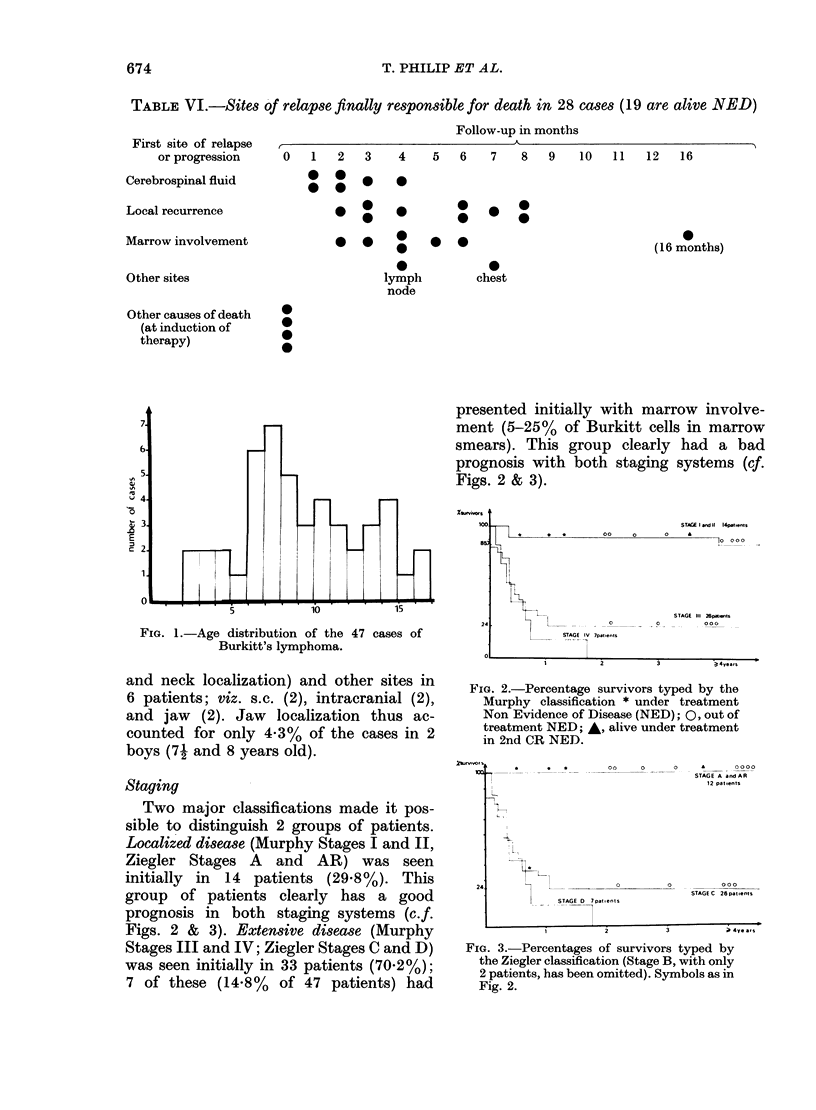

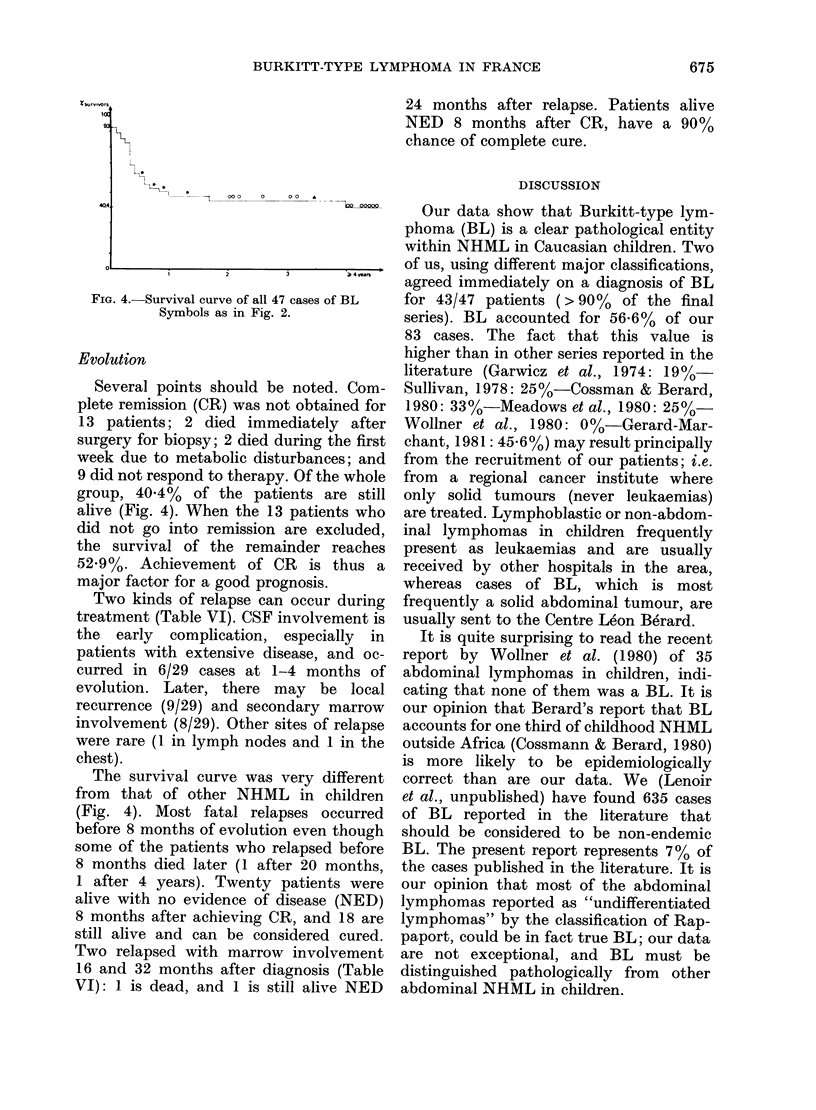

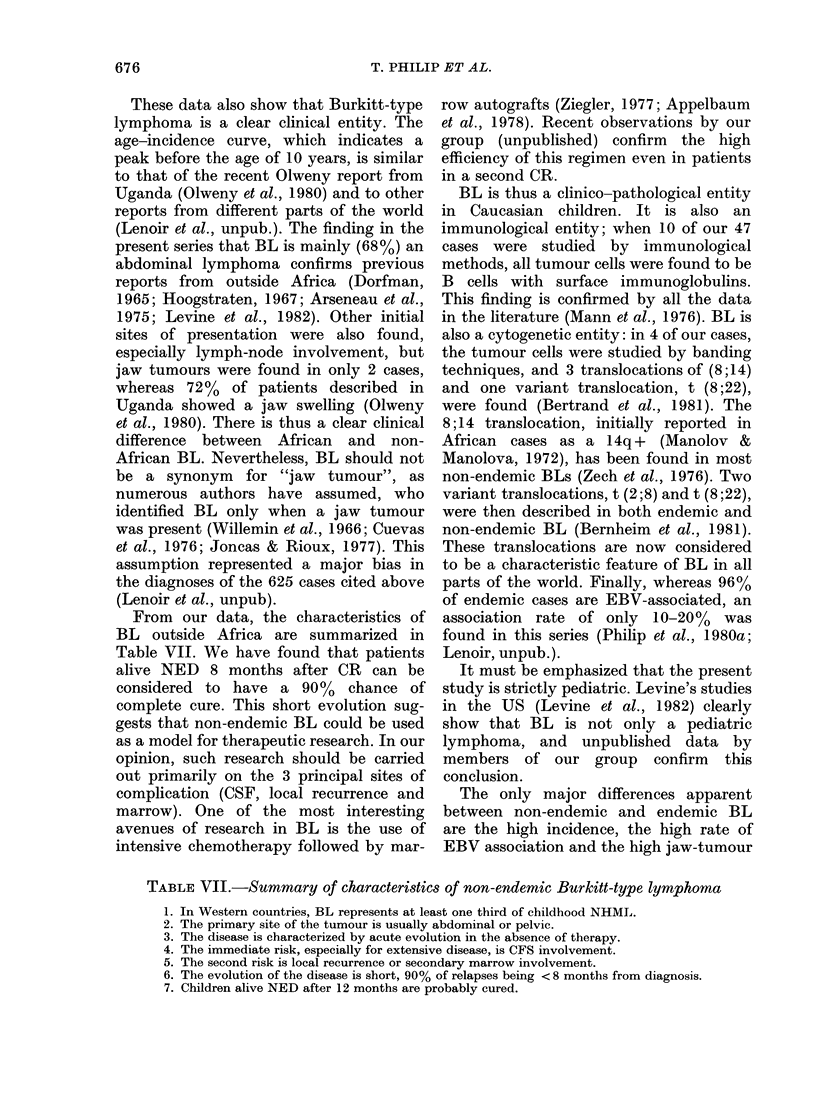

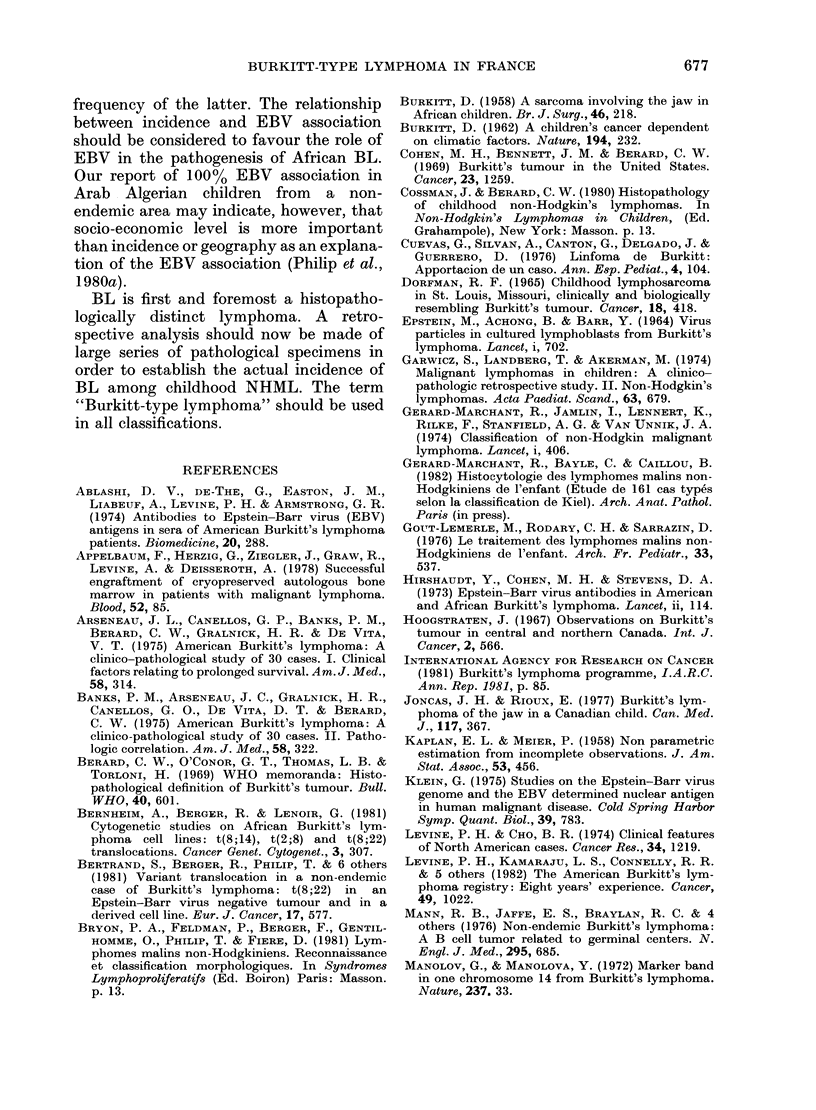

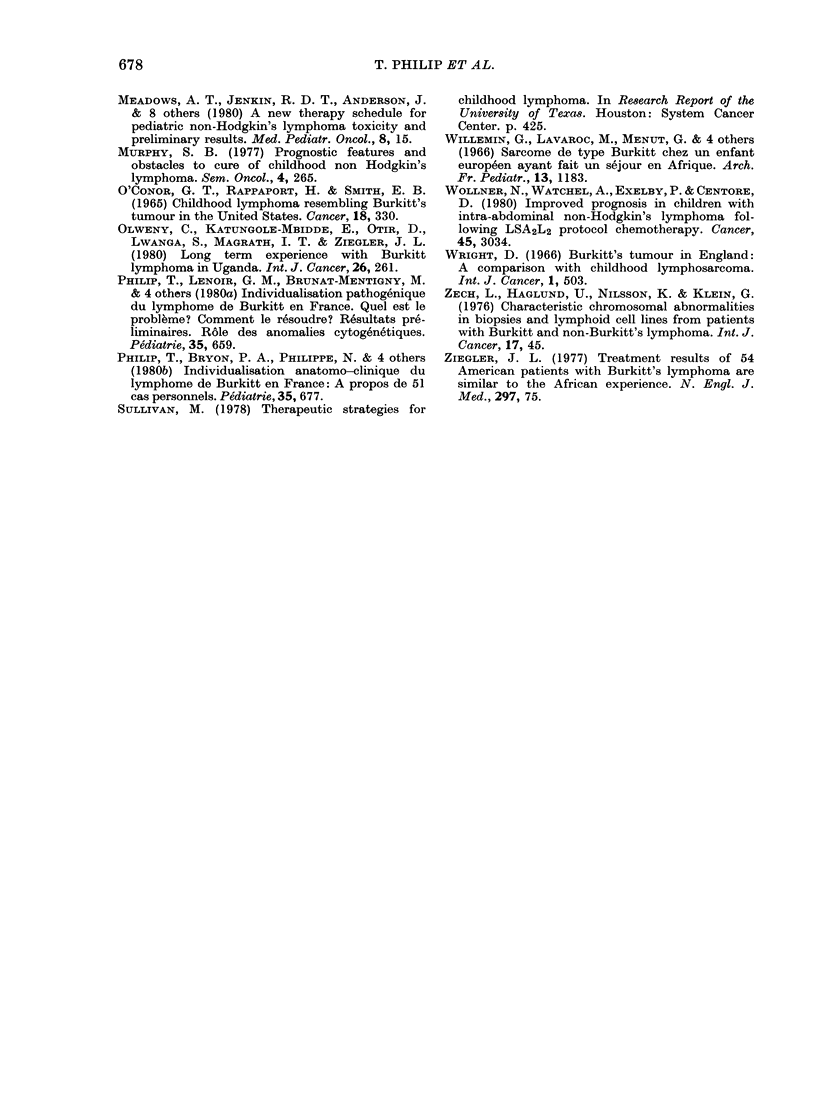


## References

[OCR_00938] Ablashi D. V., De-Thé G. B., Easton J. M., Liabeuf A., Levine P. H., Armstrong G. R. (1974). Antibodies to Epstein-Barr virus (EBV) antigens in sera of American Burkitt lymphoma patients.. Biomedicine.

[OCR_00945] Appelbaum F. R., Herzig G. P., Ziegler J. L., Graw R. G., Levine A. S., Deisseroth A. B. (1978). Successful engraftment of cryopreserved autologous bone marrow in patients with malignant lymphoma.. Blood.

[OCR_00952] Arseneau J. C., Canellos G. P., Banks P. M., Berard C. W., Gralnick H. R., DeVita V. T. (1975). American Burkitt's lymphoma: a clinicopathologic study of 30 cases. I. Clinical factors relating to prolonged survival.. Am J Med.

[OCR_00998] BURKITT D. (1962). A children's cancer dependent on climatic factors.. Nature.

[OCR_00994] BURKITT D. (1958). A sarcoma involving the jaws in African children.. Br J Surg.

[OCR_00960] Banks P. M., Arseneau J. C., Gralnick H. R., Canellos G. P., DeVita V. T., Berard C. W. (1975). American Burkitt's lymphoma: a clinicopathologic study of 30 cases. II. Pathologic correlations.. Am J Med.

[OCR_00973] Bernheim A., Berger R., Lenoir G. (1981). Cytogenetic studies on African Burkitt's lymphoma cell lines: t(8;14), t(2;8) and t(8;22) translocations.. Cancer Genet Cytogenet.

[OCR_00979] Bertrand S., Berger R., Philip T., Bernheim A., Bryon P. A., Bertoglio J., Doré J. F., Brunat-Mentigny M., Lenoir G. M. (1981). Variant translocation in a non endemic case of Burkitt's lymphoma: t (8;22) in an Epstein--Barr virus negative tumour and in a derived cell line.. Eur J Cancer.

[OCR_01002] Cohen M. H., Bennett J. M., Berard C. W., Ziegler J. L., Vogel C. L., Sheagren J. N., Carbone P. P. (1969). Burkitt's tumor in the United States.. Cancer.

[OCR_01017] DORFMAN R. F. (1965). CHILDHOOD LYMPHOSARCOMA IN ST. LOUIS, MISSOURI, CLINICALLY AND HISTOLOGICALLY RESEMBLING BURKITT'S TUMOR.. Cancer.

[OCR_01022] EPSTEIN M. A., ACHONG B. G., BARR Y. M. (1964). VIRUS PARTICLES IN CULTURED LYMPHOBLASTS FROM BURKITT'S LYMPHOMA.. Lancet.

[OCR_01027] Garwicz S., Landberg T., Akerman M. (1974). Malignant lymphomas in children. A clinico-pathologic retrospective study. II. Non-Hodgkin's lymphomas.. Acta Paediatr Scand.

[OCR_01046] Gout-Lemerle M., Rodary C., Sarrazin D. (1976). Le traitement des lymphomes malins non hodgkiniens de l'enfant. Arch Fr Pediatr.

[OCR_01052] Hirshaut Y., Cohen M. H., Stevens D. A. (1973). Epstein-Barr-virus antibodies in American and African Burkitt's lymphoma.. Lancet.

[OCR_00967] (1969). Histopathological definition of Burkitt's tumour.. Bull World Health Organ.

[OCR_01056] Hoogstraten J. (1967). Observations on Burkitt's tumour in central and northern Canada.. Int J Cancer.

[OCR_01066] Joncas J. H., Rioux E. (1977). Burkitt's lymphoma of the jaw in a Canadian child.. Can Med Assoc J.

[OCR_01076] Klein G. (1975). Studies on the Epstein-Barr Virus genome and the EBV-determined nuclear antigen in human malignant disease.. Cold Spring Harb Symp Quant Biol.

[OCR_01082] Levine P. H., Cho B. R. (1974). Burkitt's lymphoma: clinical features of North American cases.. Cancer Res.

[OCR_01088] Levine P. H., Kamaraju L. S., Connelly R. R., Berard C. W., Dorfman R. F., Magrath I., Easton J. M. (1982). The American Burkitt's Lymphoma Registry: eight years' experience.. Cancer.

[OCR_01094] Mann R. B., Jaffe E. S., Braylan R. C., Nanba K., Frank M. M., Ziegler J. L., Berard C. W. (1976). Non-endemic Burkitts's lymphoma. A B-cell tumor related to germinal centers.. N Engl J Med.

[OCR_01111] Murphy S. B. (1977). Prognostic features and obstacles to cure of childhood non-Hodgkin's lymphoma.. Semin Oncol.

[OCR_01121] Olweny C. L., Katongole-Mbidde E., Otim D., Lwanga S. K., Magrath I. T., Ziegler J. L. (1980). Long-term experience with Burkitt's lymphoma in Uganda.. Int J Cancer.

[OCR_01137] Philip T., Bryon P. A., Philippe N., Souillet G., Freycon F., Gérard-Marchant R., Brunat-Mentigny M. (1980). Individualisation anatomo-clinique du lymphome de Burkitt en France. A propos de 51 cas personnels.. Pediatrie.

[OCR_01013] Pineda Cuevas G., Alvarez Silván A., García Cantón J., Fernández Delgado J., López Guerrero D. (1976). Linfoma de Burkitt. Aportación de un caso. An Esp Pediatr.

[OCR_01153] Wollner N., Wachtel A. E., Exelby P. R., Centore D. (1980). Improved prognosis in children with intra-abdominal non-Hodgkin's lymphoma following LSA2L2 protocol chemotherapy.. Cancer.

[OCR_01160] Wright D. H. (1966). Burkitt's tumour in England. A comparison with childhood lymphosarcoma.. Int J Cancer.

[OCR_01172] Ziegler J. L. (1977). Treatment results of 54 American patients with Burkitt's lymphoma are similar to the African experience.. N Engl J Med.

